# Barriers to teaching evolution in higher education

**DOI:** 10.1186/s12052-021-00151-1

**Published:** 2021-08-13

**Authors:** Ethan R. Tolman, Daniel G. Ferguson, Gabriella Hubble, Mahealani Kaloi, Megan Niu, Jamie L. Jensen

**Affiliations:** 1 Department of Plant and Wildlife Sciences, Brigham Young University, 4125 LSB, Provo, UT 84602 USA; 2 Department of Biology, Brigham Young University, 4102 LSB, Provo, UT 84602 USA; 3 Department of Biology, Brigham Young University, 4102 LSD, Provo, UT 84602 USA; 4 Department of Neuroscience, Brigham Young University, S192A ESC, Provo, UT 84602 USA

**Keywords:** Evolution, Higher education, Barriers, Faculty development, Religion, Ecological model

## Abstract

**Background:**

Although progress has been made in evolution education, many educators face significant barriers in their efforts to teach evolution specifically, and science in general. The purpose of this study was to identify faculty-perceived barriers to teaching evolution, specifically in religiously affiliated institutions or institutions with a highly religious student body, as well as resources faculty feel would help promote discourse around faith, evolution and science. To do this, we held a workshop with teams consisting of a science professor, a theologian and a pastor (of the predominant on-campus faith tradition) from 17 different institutions of higher education with highly religious student bodies for the purpose of helping them to create a curriculum to address perceived conflicts between science and faith. During the workshop, participants created posters identifying barriers they face and resources they perceive as helpful. These posters were analyzed for prevalent themes and framed within an ecological model of behavior.

**Results:**

These teams identified prevalent barriers at each level of the ecological model. Intrapersonal factors included a fear of rocking the boat and a fear of student conflict. Interpersonal factors included perceived student lack of knowledge, student ideology, and student apathy. Institutional factors included work politics, a lack of relevant discourse surrounding the conflict, and mixed messaging to students. Community factors included social norms associated with various student demographics. And public policy factors included local and state government attempts to limit the teaching of evolution. Additionally, participants identified resources that they felt would facilitate overcoming conflict including colleagues as change agents, various assets, and tools to negate conflict.

**Conclusions:**

We determined that many of the concerns are addressable, and many resources are attainable. We urge the community to work toward these solutions. Additionally, we compare our findings to what the literature has shown and discuss the implications of faculty perceptions as compared to the published literature.

**Supplementary Information:**

The online version contains supplementary material available at 10.1186/s12052-021-00151-1.

## Background

Evolutionary theory remains a seemingly controversial topic in the eyes of the public in the United States and other countries around the world (Pew Research Center 2019). Evolution, most broadly defined, is descent with modification, but can be more narrowly defined as a change in the genetics of a population over time (Reece et al. 2011). In investigating evolution acceptance, many researchers find it helpful to distinguish between evolution within populations over a short period of time (termed ‘microevolution’) from speciation and divergence that occur as a result of evolutionary processes over a longer period of time (termed ‘macroevolution’) [e.g., (Nadelson and Southerland 2012)]. This is because public acceptance of these concepts differ, sometimes significantly [e.g., (Alters and Alters 2001; Miller 2008; Scott 2009)]. In addition, we can go further and identify specifically human evolution as yet another category of which public perceptions markedly differ [see (Nadelson and Southerland 2012)], and of which acceptance levels are the lowest (Inc G 2007). A Gallup poll from 2017 found that 38% of Americans still hold strict creationist beliefs in regards to human evolution (Inc G 2017); there is significant variation among estimates, however, depending on how the survey was worded (Pew Research Center 2019). Additionally, lack of evolution acceptance appears to be correlated with distrust of the scientific community at large. 29% of adults in the United States believe that scientists do not agree that humans have evolved over time, and among those adults who believe that humans and other life forms have existed in their current form since the beginning, an astounding 46% believe that scientists do not agree that humans have evolved over time (Pew Research Center 2015). This is despite the fact that scientists themselves are largely in consensus regarding human evolution with 97% accepting that humans have evolved over time (Pew Research Center 2015). This skewed perception of scientific consensus regarding evolution, and perhaps misunderstanding regarding the scientific process generally, can have potentially serious and far-reaching effects in the realm of public policy regarding vaccinations, space exploration, the approval of new technologies, and our response in the face of global issues such as the COVID-19 pandemic (National Science Teaching Association 2020; Moore and Cotner 2009; Augustine 1998; Reid 2020).

While the issue of evolution acceptance is complex, it appears that a significant contributing factor to the current low acceptance rate is a lack of training and appropriate teaching within the public education system (Berkman and Plutzer 2012). Many teachers enter the workforce without adequate training and knowledge of evolutionary concepts, and without the skills to firmly present scientific data to perhaps initially skeptical students (Berkman and Plutzer 2012; Hawley and Sinatra 2019). Thus, teachers lack the necessary resources to address the conflicts they will encounter with students. Additionally, increasing students’ understanding of the mechanics of evolutionary theory alone has yielded mixed results in regard to promoting evolutionary acceptance (Sinatra et al. 2003; Mead et al. 2017). This can be particularly alarming to those within the scientific community, as evolution is generally viewed as one of the core and unifying concepts within the sciences (National Science Teaching Association 2020).

A significant challenge to increasing evolution acceptance is that students enter the higher education system with many prior conceptions. These conceptions are highly influenced by their religiosity, parental views, and information received in high school from science teachers, as will be explained by the following research. Religiosity has been found to be one of the strongest indicators of rejection of evolution, both human evolution and general evolutionary theory (Heddy and Nadelson 2012). As for parental influence, Winslow (2011) found that parents have a strong impact on children’s acceptance of evolution. When given pre- and post-open-ended surveys of their beliefs on evolution, many students in an evolution course claimed that it was an “easier route” to reject evolution, because it avoided tension at home. Students also expressed that they had anxiety about talking with their parents about evolution. In regard to high school education, Moore and Cotner (2009) found that evolution and creationist views of first-year college students were strongly associated with what information was given to them in high school, and how that information was presented. All of these factors together present professors with a challenge through which they may not be adequately prepared to navigate. In other words, the lack of adequate resources to help teachers effectively communicate with students on this sensitive issue can present a barrier to acceptance.

While many educators have a desire to help their students become more accepting of evolutionary theory (Barnes and Brownell 2016), there are additional factors that have the potential to confound the difficulties already inherent in teaching evolution. Discourse surrounding science and faith is known to increase undergraduate acceptance of evolution (Manwaring et al. 2015; Barnes and Brownell 2016; Lindsay et al. 2019; Tolman et al. 2020), but often requires institutional change. Such institutional change likely necessitates the availability of a large number of resources. For example, Sunal et al. (2001) found that amongst science professors striving to make positive change in their teaching and at their institution, administrative and collegial support was necessary for 90% of participants. Administrative presence, effective goal setting and planning, communication with others who had a similar goal, and interpersonal skills were additional factors important for positive change. In addition to institutional characteristics, the ideology of a professor can serve as a major barrier to the promotion of discourse around science and faith, as many professors do not believe their job is to encourage students to accept evolution, or do not themselves believe that science and faith are compatible (Barnes and Brownell 2016). Overcoming biases and improving teaching requires professors to engage in extensive reflection on their teaching—a task for which not all faculty have the time (Kreber 2004,2005).

Although there has been ample research about evolution acceptance and education, there is much we do not know about the experience of faculty who teach evolutionary theory, especially to highly resistant audiences, and especially about the factors that influence their decisions on how to approach the topic of evolution. In this study we survey ministers and biology and theology professors at highly religious institutions to determine what factors they perceive to influence institutional discussion surrounding science and faith, specifically regarding the teaching of evolution, and what resources they feel would be helpful. Because these faculty come from a wide range of institutions, all with highly religious students, and are representative of the general faculty at most schools (i.e., they are not experts in evolution education), they provide a unique and valuable insight into the lived experiences of those striving to teach evolution in a potentially hostile environment. They are examples of the end-users for which the research is striving to provide evidence-based guidance. Thus, their insights are critical to the problem at hand.

## Theoretical framework

**Fig. 1:**
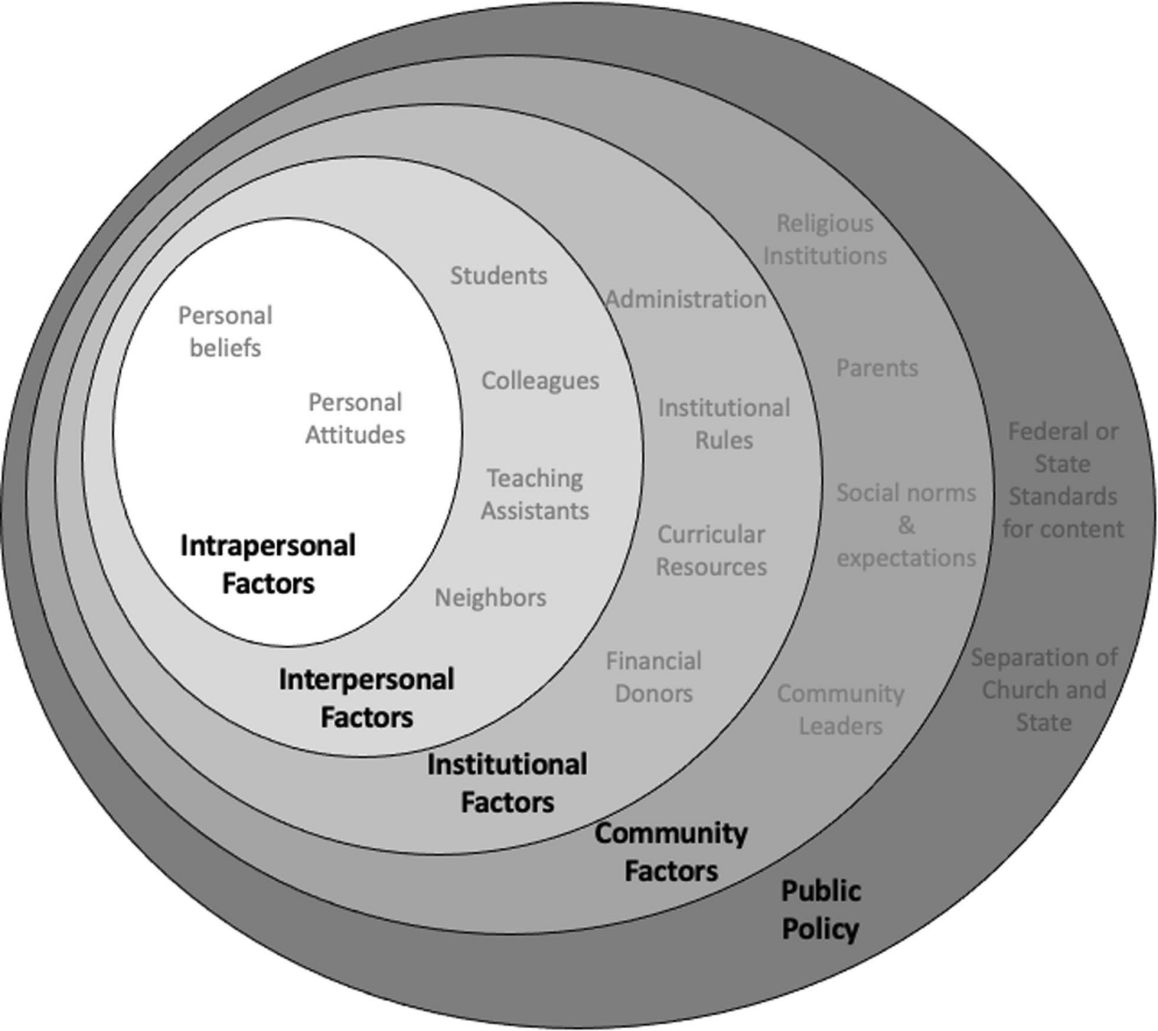
The Ecological Model of Behavior regarding the teaching of evolution in the undergraduate classroom

In the management and study of health behaviors, a common theoretical framework that frames human behavior is the Ecological Model of Behavior [see summary in Sallis et al. (2008)].The ecological model considers the fact that humans exist in a complex ecosystem with multiple levels of influence that govern behavior and choice. We see university faculty existing in a similar ecosystem in which multiple factors play a role in the way they view the conflict between evolution and religion and the way that they approach solutions to this conflict. We have adapted the ecological model for our purpose in characterizing factors influencing faculty attitudes and behavior and illustrated it in Fig. 1.

In the ecological model, the innermost level of influence is the intrapersonal or individual factors, which includes one’s own beliefs, knowledge, and attitudes toward the topic. In this case, it would be the individual faculty member’s own struggles with the perceived conflict and the way in which they believe resolution is best accomplished. It would also include their beliefs about the institutions, the climate, and their own students.

The second level is the interpersonal factors, or interactions with people around them that can either hinder or help promote a given behavior. In our framework, this would include interactions with students in the classroom, with colleagues in the hallways, with neighbors at church, etc.

The third level is the institutional or organizational level that might include any rules, policies, or institutional structures that place weight on an individual’s decisions. In our framework, these factors would include administrative pressures to teach or not teach a controversial topic, pressures from funding bodies of the institute or other financial donors, pressures from within a department by administrators who hold their own opinions or agendas on the way in which the topic of evolution should be treated, and any curricular resources that are provided or forbidden.

The fourth level includes community factors such as social norms or expectations that may exist within a community and may be very different between communities with which the individual interacts. In our framework, these community factors would most certainly involve religious institutions that may put pressure on university faculty to avoid the conflict. It would also include pressure from parents of students or other, non-institution-affiliated individuals who voice opinions about the way in which evolution should be taught.

The last level is public policy that would include any local, state, or federal policies or law that regulate a behavior. In our framework, this would include any state or federal standards for content that must be taught for accreditation purposes. It would also include the common pressure that university faculty feel with maintaining a separation of church and state in their teaching.

By sampling our participating teams, we are getting insights into the influences they are currently experiencing when it comes to discussing issues of science and faith. We frame our findings using the ecological model of behavior (specific to faculty perceptions only) and compare their responses to what is known about institutional change and teaching evolution in higher education in order to reach our goal of clarifying the main influences of institutional discussion surrounding science and faith.

## Methods

### Recruitment

Recruitment fliers were sent to the biology faculty at institutions of higher education where students might face conflict between religious influences and learning evolutionary science. These institutions were identified due to either the religious affiliation of the institution, or a highly religious student body. Potential participants were required to come in a team of three that included (1) a faculty member from a biology-related discipline who teaches evolution in their undergraduate biology course, (2) a faculty member from the same institution from a theology-related discipline who can speak to the predominant faith traditions of students, and (3) a local minister representative of the majority of the student body from this same institution. No prerequisites for any expertise in teaching evolution was required. In essences, these teams were intended to be representative of the general population of biology and theology faculty that typically reside at institutions across the country. Teams were invited to attend a 3-day workshop in which they would be co-authoring learning materials that could be shared broadly and that are specific to a faith tradition to offer students a way to reconcile faith and evolution without promoting or degrading religion. The focus of these curricular materials included macroevolution, microevolution, and human evolution. All travel and lodging expenses were covered, and additional stipends were made available for participation in follow-up activities related to the research.

### Sample

Participating teams represented a diverse sample of religious academic institutions from several religious affiliations, locations in the country, Carnegie classifications, size, and public or private classifications, as summarized in Table 1. There were a total of 17 academic institutions participating, each having a team consisting of a science professor, theology professor and minister. 15 institutions chose to answer our prompts.

**Table 1:** Summary of participating institutions (numbers of institutions are indicated in parenthesis)

Religious affiliation	Location	Carnegie basic classification	Student population	Public or private
Non-denominational Christian (5)	Southern USA (4)	Doctoral University (6)	10,000 + (4)	Private not-for-profit (16)
Catholic (3)	Midwestern USA (5)	Master’s College and University (5)	5000–10,000 (3)	Public (2)
Jewish (2)	Southwestern USA (4)	Baccalaureate College (5)	2000–5000 (5)	
Non-affiliated (3)	Northeastern USA (2)	Associate College (1)	< 2000 (6)	
Assemblies of God (1)	Eastern USA (1)	N/A (1)		
Presbyterian (1)	Hawaii USA (1)			
Nazarene (1)	International (1)			

### Workshop

The purpose of the workshop was to help institutional teams overcome barriers to discussions surrounding evolution and religion at their respective institutions. It was facilitated by a team of experts in evolution, evolution-specific pedagogy, and theological issues related to evolution. Specifically, our team consisted of a discipline-based education researcher in biology who specializes in reconciling evolution and religious beliefs; an evolutionary biologist who has done extensive work in evolution acceptance; a biologist and discipline-based education researcher with extensive experience in teaching evolution to religious audiences; a theologian who specializes in biblical interpretation, ecclesiology, and the intersection of science and theology; and a physicist with extensive experience in science and religion communications. The workshop began with an introductory presentation and discussion about the importance of scientific literacy and the effectiveness of a reconciliatory approach to teaching evolution that allows students room to embrace evolution while maintaining their religious identities. We then shared data outlining the problem (i.e., low acceptance rates) and supporting the effectiveness of this approach as collected from the workshop facilitators’ institutions. We discussed potential stumbling blocks for students and debunked the warfare model of science versus religion. We then had teams participate in an interactive activity on the nature of science and both its uses and limitations. We then shared two specific case studies of approaching reconciliation at religious institutions. By reconciliation, we mean that we used an approach that allowed students to embrace evolutionary theory while maintaining their religious beliefs, in other words, to allow students to find a way to reconcile their religious beliefs with the science they are learning [see (Lindsay et al. 2019), for an example of this methodology and evidence of its success]. These case studies highlighted the barriers, both personal barriers of how to reconcile religious faith with science and cultural barriers due to the religious beliefs and histories that our students brought to the classroom, that we had experienced and the ways in which we had overcome these barriers, one from the perspective of the Church of Jesus Christ of Latter-day Saints and one from a Nazarene tradition. In our discussion with participants, we defined evolution as the full theory, including microevolutionary concepts, macroevolutionary concepts, and human evolution concepts. We did not limit discussions of conflict to only one aspect of the theory and allowed teams to consider what the conflict between religious faith and evolution looked like for them with their student body. We asked them to describe their perspective on the current state of discourse surrounding these issues on their campus at various levels. In our discussions with participating teams, we aimed to clearly reveal the main influences at play in their institutions regarding these discussions. To do this, we asked a series of questions specifically targeting perceived barriers and influences. First, we asked them to identify, from their perspective, factors that influenced this discourse, both those involving the individuals having the conversation directly in the classroom (i.e., the students and faculty), what we referred to as “internal factors”, and those involving factors outside of the direct classroom, such as the institutional religious affiliation or the community involvement in institutional matters, what we referred to as “external factors”. All responses were based on personal experiences at their institutions and therefore represent their perspectives. We also asked them about their greatest challenges to reconciliation and what resources they felt would be most helpful in overcoming these challenges. The discussion was open and flexible and no format was provided for their responses allowing them to use whatever form participants needed to express their views. The prompts displayed on screen to them were as follows and were all in relation to discourse surrounding evolution:

- “Describe the general state of discourse about science and faith (especially regarding evolutionary science) at your institution among faculty, students, in the classroom, etc.
- What internal (e.g., student or faculty religiosity) and/or external factors (e.g., institutional religious affiliation) influence this discourse?
- What are the greatest challenges to the reconciliation of evolution and faith at your institution?
- What are the most important resources at your institution for nurturing healthy discourse?
- What additional internal or externally supported resources can you imagine would support this?”

Participants were asked to write their responses on a posterboard to illustrate their collective answers to these prompts and have them in a format that was easily shareable with other groups. Posters were then analyzed for themes using the procedure described below in “Coding”.

### Coding

Posters were analyzed individually for each of three a priori categories, dictated by the prompts given to participants: internal factors, external factors, and resources. Within each category, posters were analyzed for emergent themes, or factors, using the strategy outlined by Charmaz (2014). Briefly, emergent coding was done by three individual raters who analyzed all posters and created themes that could be used to effectively group responses on posters into identifiable categories. The raters met together and discussed themes to come to a consensus on a coding rubric (Table 2), in which all responses fit a category and no new categories were emerging. Each poster was re-analyzed to fit responses into the coding rubric until all 15 posters were coded. We used emergent coding to avoid biasing our interpretations toward any given framework so that we truly captured what faculty were expressing. Once themes emerged, they were compared to the ecological theoretical framework to determine at what level these factors were acting; this framework was used to attempt to better understand how these themes may be affecting faculty behavior as they approach these difficult discussions surrounding evolution and faith.

**Table 2:** Coding guide

Coding category	Ecological framework level	Subcategories	Example quotes
Internal factors			
Fear of rocking the boat	Intrapersonal	Not wanting to subvert religious authority, Professors fear conflict	“[The] greatest challenge to reconciliation [is] fear of compromise [because of] biblical authority, cultural Authority [or] Religious Authority.”
Fear of conflict (students)		Fear among students, perceived conflict on the part of students	“Students … operate out of perceived conflict between faith and science”
Student ideology	Interpersonal	Political views, fundamentalism, unwillingness of students to change opinion	“Move toward ultra-orthodox, [students see] evolution as secular!”
Student lack of knowledge		Religious misconceptions, scientific misconceptions, misunderstanding of the nature of science, believing evolution promotes racism	“Cultural inertia, misinterpretation of the teachings about the creation, human origins and relationship between evolution and ‘creation’ of humans.”
Student apathy		Specifically list student apathy	“Friendly-apathetic [attitude]”
External factors			
Politics	Institutional	Institutional or governmental politics, lack of unity, fear of losing job, institutional stance is unclear, external stakeholders, church stance is incompatible with evolution	“Fear is a great challenge to any reconciliation. Fear of losing faith or one’s job.”
Mixed messaging to students		Lay ministry, curricular issues, evolution is not thoroughly covered, faculty in conflict with each other	“Faculty feels they have no time to teach evolution; they must cover what the curriculum requires.”
Lack of discourse		Specifically listed	“Unless issues are raised, no active discourse, don’t ask, don’t tell”
Demographics	Community	Anything about student demographics	“75 different nationalities represented in the student body”
Resources			
Faculty as a force for change	Interpersonal	Faculty are strong role models, supportive faculty, professor accessibility, faculty training	“Faculty talks on their personal faith and scholarly journey”
Assets	Institutional	Money, technology, books	“There is a need for new discourse including books and materials”
Negating conflict		Compatible theology, visiting authorities, non-resistant administration	“VP for spiritual development has budget to bring science and faith speakers to campus twice annually”
Other institutions		Partnerships with other institutions	“There is a need for new discourse…perhaps [including an] external partnership with [a] more experienced institution.”

### Qualitative analysis

For each barrier and resource category identified, we selected a quote we felt was representative of responses and described our interpretation. We have included images of all posters as Additional file 1.

### Quantifying results

In an effort to summarize our limited data for the reader, we calculated what percentage of teams listed at least one factor in each subcategory. We also took the sum of all individually listed internal factors, and determined what percentage fell into each subcategory. This process was repeated with the external factors and resources. If the same statement was mentioned more than once on a particular poster, the subcategory was only coded for once. If two different statements within the same subcategory were mentioned, the subcategory was coded for twice.

## Results

### Internal factors

In response to the prompt, ‘What internal factors influence this discourse?’, we identified five main factors that all fall within the first two levels of the ecological model. Intrapersonal conflicts included a “fear of rocking the boat” and a fear of perceived conflict with students. Interpersonal barriers included entirely perceived student attributes including ideology, a lack of knowledge, and apathy toward the issue. Regarding faculty fear of rocking the boat, one team wrote on their poster that the “greatest challenge to reconciliation [is] fear of compromise [because of] biblical authority, cultural authority [and] religious authority.” This statement demonstrates that these faculty members are afraid to encourage students to reconcile their faith with science because they were afraid to suggest a compromise to what students see as authoritative. In other words, they were afraid to challenge the established social or cultural norms. Thus, while this was an intrapersonal belief (a fear of pushback, so to speak), it drew upon both institutional and community factors from which faculty perceived that pushback arises.

Several teams expressed a second intrapersonal fear in that they did not attempt to encourage students to reconcile faith and science because they believed that students felt uncomfortable with the conflict. One team confirmed, “Students…operate out of perceived conflict between faith and science.” Here the participants make it clear that they perceive that their students believe faith and science to be in direct conflict. Again, while this is an intrapersonal belief, it stems from large ecological factors including the cultural environment (community) and the students themselves (interpersonal).

All interpersonal factors centered around concerns with perceived student characteristics. A representative sample of student ideological concerns was seen by one team that described teaching evolution as being difficult because their students have made a “move toward ultra-orthodox, [viewing] evolution as secular!” These educators expressed their feeling that their students are moving towards a more orthodox view of their faith. This ideological barrier leads students to feel that evolution is secular, making reconciliation more difficult. Political ideology was also mentioned. Many teams indicated that they thought students lacked accurate knowledge of evolution, of religious teaching, or of both and that they simply misinterpret the ways in which these disciplines might intersect. One team wrote, “Cultural inertia, misinterpretations of the teachings about the creation, human origins and relationship between evolution and ‘creation’ of humans.” Lastly, some teams felt that a major barrier to reconciliation was that students simply do not care. One team wrote, “friendly-apathetic (attitude)”. All of these factors would be considered interpersonal as they deal with faculty interactions with students. However, these factors are certainly influenced by community factors outside of the institution (e.g., religious groups, parents, and social norms).

**Fig. 2:**
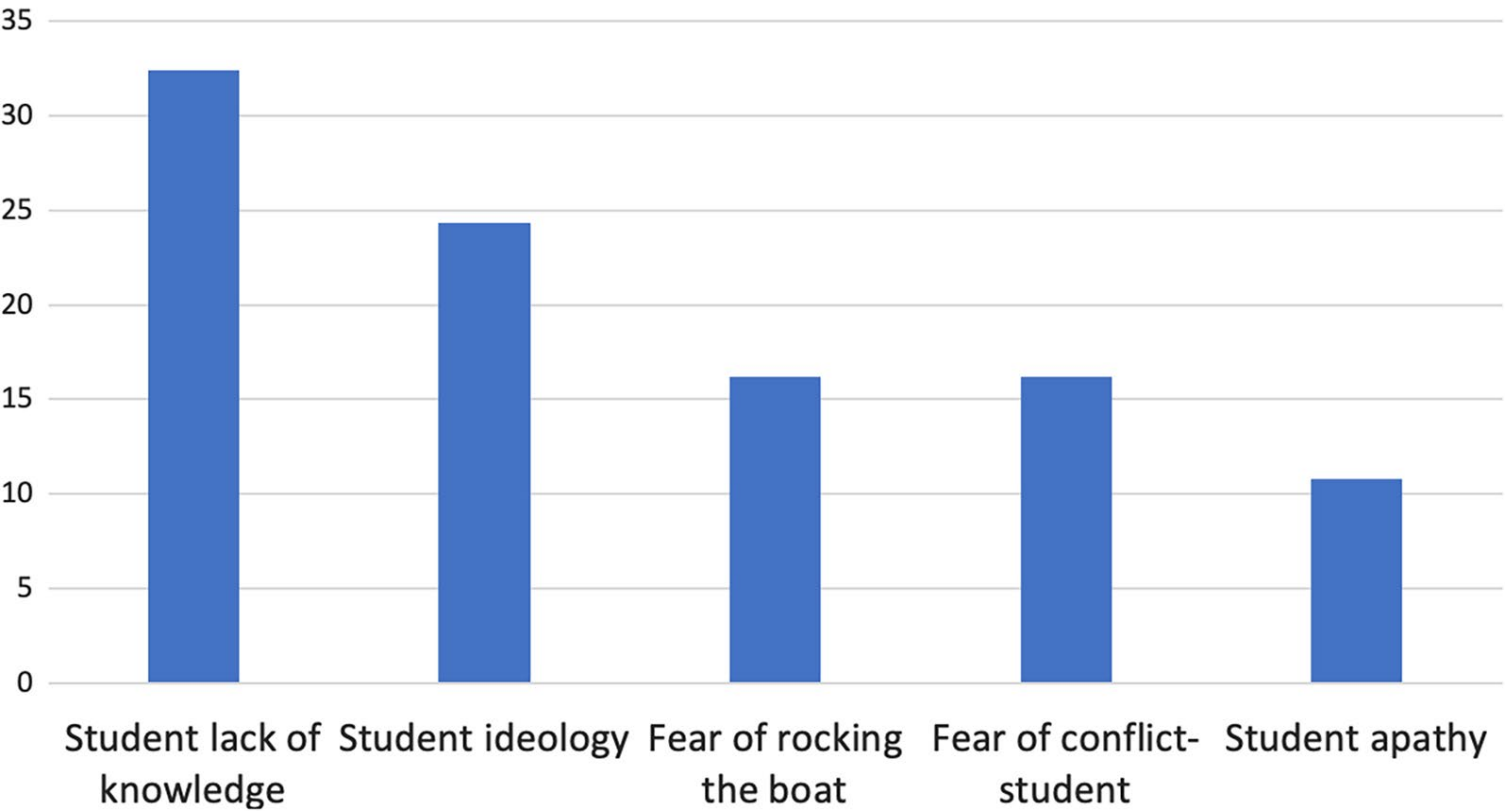
The percentage of internal factors influencing discourse around faith and science by category. Student lack of knowledge was the most commonly listed category, containing over 30% of the listed internal factors

Given the total list of internal factors cited by all teams combined (many teams reported multiple categories of factors, and multiple factors within a single category), student lack of knowledge was the most common concern among the internal factors (32.4%), followed by student ideology (24.3%), fear of rocking the boat (16.2%), and student apathy (10.8%); see Fig. 2.

### External factors

In response to the prompt, ‘What external factors influence this discourse?’, we identified four factors related to the second two levels of the ecological model. Institutional factors included work politics, a lack of discourse, and mixed messaging to students from administration; the community factor identified related to specific student demographics that would influence the norms and expectations of students. In regard to work politics, one team wrote, “Fear is a great challenge to any reconciliation. Fear of losing faith or one’s job.” Mentioning that they might lose their job over this is an extreme example of an institutional political factor posing a barrier to a faculty’s desire to teach reconciliation. Other examples extended to the public policy level by mentioning efforts by local and state governments to limit the teaching of evolution in public schools.

Many teams referenced issues relating to a lack of discourse at the institutional level. One team wrote, “Unless issues are raised, no active discourse, don’t ask, don’t tell.” Here the team describes a culture with little discourse around science and faith, which serves as a barrier to promoting reconciliation. This was a fairly common sentiment. Some teams also expressed concern with the mixed messaging to students. As an example, one team wrote, “Faculty feels they have no time to teach evolution; they must cover what the curriculum requires.” Issues with the curriculum were all coded into the mixed messaging category. In describing their curriculum lacking evolution, they are implying that it is not an important topic. Other examples of mixed messaging included negative messages about evolution that students received at church (extending barriers to the community level) or in respective theology classes at the same institution where they were receiving biology instruction. Lastly, some teams remarked on a specific student demographic that posed a challenge. One team stated, “75 different nationalities represented in the student body.” The team expressed that this diversity makes it difficult to create reconciliation methods that would be appropriate to the full student population given that each student may be coming from a community with different norms and expectations. Contrary to this example, but still within this category, one institution remarked that a homogenous student body posed a barrier to reconciliation.

**Fig. 3:**
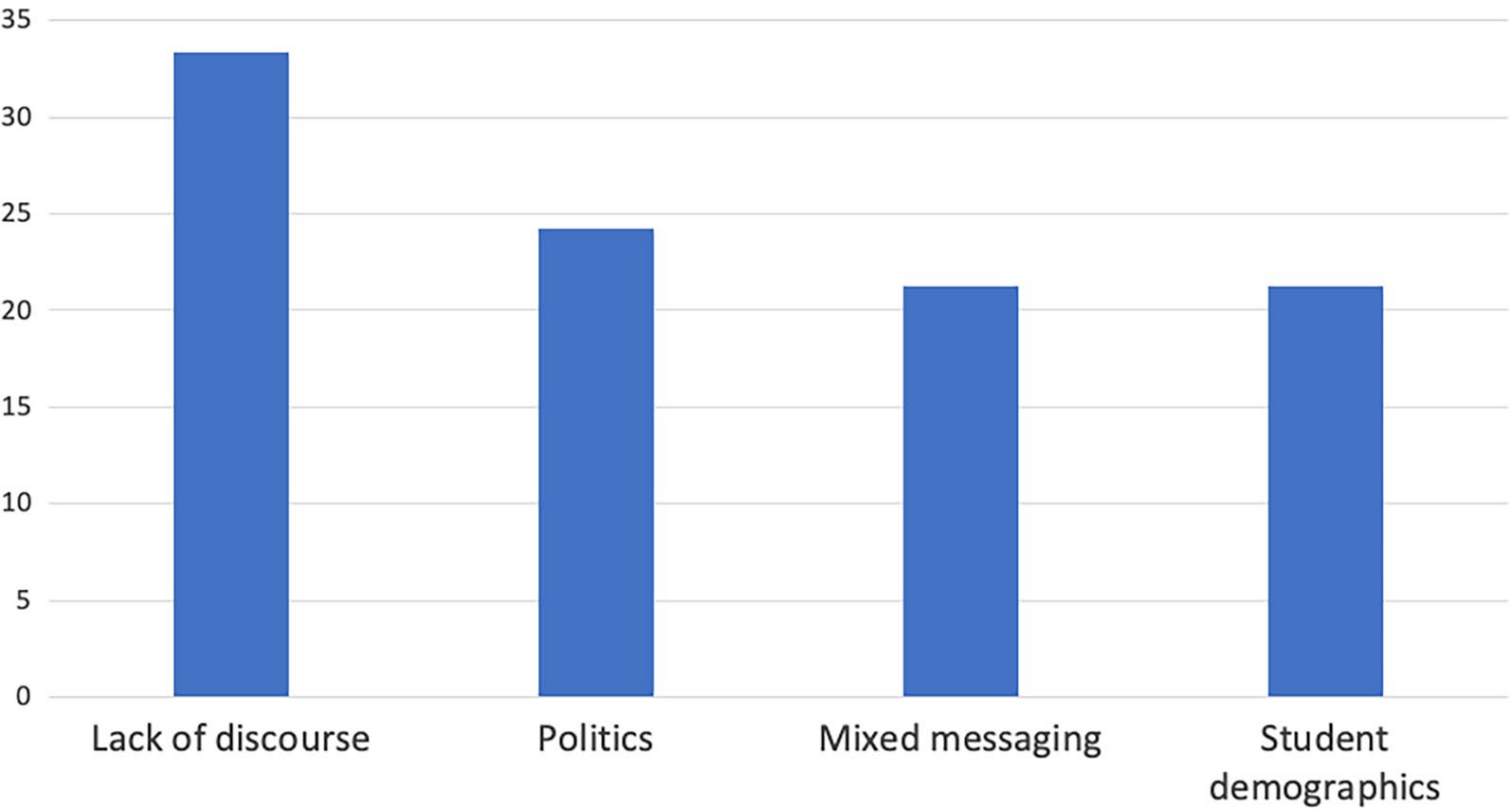
The percentage of external factors influencing discourse around faith and science by category. Lack of discourse was the most commonly list category, containing over 30% of the listed external factors

We combined all external factors listed by all institutions. Of the four external factor categories considered, 33.3% fell under lack of discourse, followed by politics (24.2%), mixed messaging (21.2%), and student demographics (21.2%) (Fig. 3).

### Resources

In response to the prompt, ‘What additional internal or externally supported resources can you imagine would support this?’, we identified four categories for the resources that participating teams felt would be helpful in nurturing healthy discourse surrounding faith and evolution. These categories fell into both an interpersonal and institutional level. Within the interpersonal category was faculty as a force for change; institutional-level factors included assets (e.g., curricular materials, books, resources), ways to negate conflict, and other institutions. In regard to faculty as a force for change, one team suggested a potential resource could be “faculty talks [i.e., recorded speeches] on their personal faith and scholarly journey.” These participants believed that faculty sharing their personal journeys could be a valuable resource for encouraging reconciliation. This is an encouraging trend that the faculty members felt that they can directly act as a resource at their own institutions. Teams commonly expressed that faculty could be a force for change by serving as role models and changing institutional culture.

By assets, many teams mentioned books, talks, and other resources that could be helpful in promoting these conversations. One team said, “There is a need for new discourse including books and materials.” Other examples of assets included money and new technologies for teaching. Related to this were specific calls for curriculum related to this intersection. One team wished for unity “related to evolution/science and the gospel [through] curricular alignment.” This particular team shared a story of how aligning their curriculum has brought about unity in their department when it comes to science and “the gospel”. This sentiment was expressed by several institutions that suggested courses that address science and faith, and curricular unity between religion and science courses.

Several teams suggested explicit interventions to help negate the conflict perceived by students. One team mentioned that their “VP for spiritual development has budget to bring science and faith speakers to campus twice annually” and that this can alleviate conflict. This is just one example of a way in which their institution actively negates conflict between science and faith. Other resources included encouraging the teaching of compatible theology, having a non-resistant administration, and encouraging open dialogue. And one team mentioned partnerships with other institutions as being a valuable resource. They stated, “There is a need for new discourse…perhaps [including an] external partnership with [a] more experienced institution.”

**Fig. 4:**
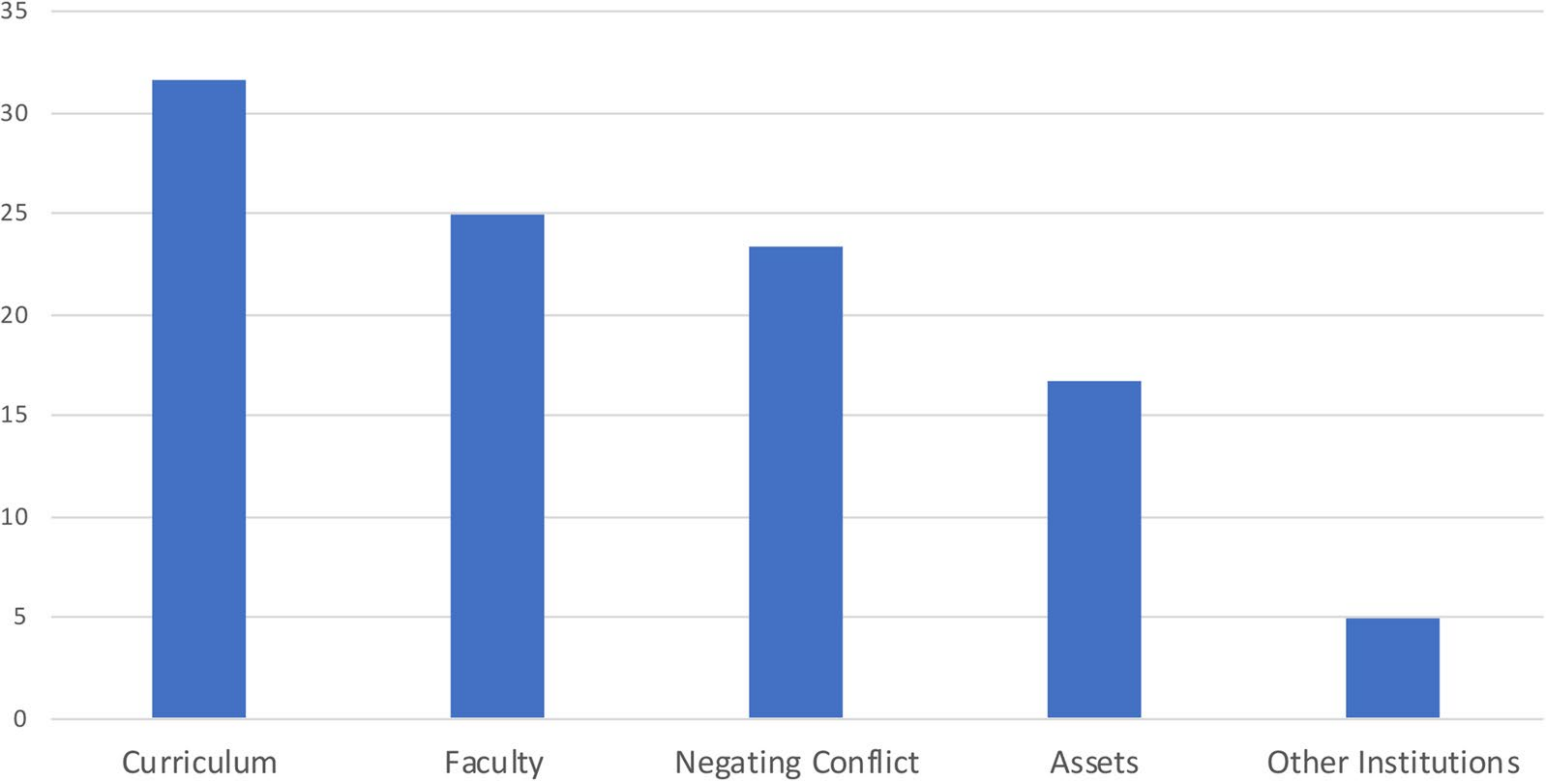
The percentage of proposed resources teams felt would promote discourse around faith and science, and aid in the teaching of evolution. Curriculum was the most commonly listed category, containing over 30% of the listed resources

Combining all proposed resources from all participating teams, curriculum was the most commonly suggested resource (31.7%), followed by faculty as a force for change (25.0%), negating conflict (23.3%), other assets (16.7%) and other institutions (5%) (Fig. 4).

## Discussion

Faculty participants at our workshop identified factors at all levels of the ecological model that they perceive as barriers to teaching evolution, especially among highly religious students. Many of the barriers perceived within lower levels of the model are likely highly influenced by factors at higher levels, e.g., barriers involving student conflict likely arise from community norms and expectations brought about by religious influence. Faculty participants also identified potential resources at both the interpersonal and institutional levels of the model.

### Internal factors

The primary internal factors perceived by professors and ministers to hinder student acceptance of evolution were at both the intra- and interpersonal levels. The highest cited factors were both pertaining to perceptions of students (interpersonal): student lack of knowledge and student ideology (Fig. 2), both of which would be highly influenced by community factors. This perception aligns with the previous idea that religious affiliation appears to be a significant predictor of acceptance levels (Charmaz 2014), which is heavily supported in the literature (Mazur 2004; Evans 2011; Keeter et al. 2012; Baker 2013). Some studies have shown that religious students are less likely to accept and understand evolution (Manwaring et al. 2015). Winslow et al. (2011) also found that in order to reconcile evolution with religious beliefs, students had to desire to develop a positive relationship between religion and science within their worldview. This emphasizes that student ideology may indeed be one of the critical predictors of evolution acceptance necessitating approaches that ease the tension between student ideology and the science of evolution. While addressing students’ lack of knowledge can increase acceptance of evolution, it remains ineffective if students are unwilling to change their mind (Winslow et al. 2011). Additionally, other studies have shown that addressing purely scientific misconceptions regarding evolution may be insufficient to increase evolutionary acceptance, even when those measures are otherwise successful in teaching evolutionary concepts (Sinatra et al. 2003; Walter et al. 2013; Rios et al. 2015; Dunk et al. 2019). Even though many of the professors and ministers viewed student lack of knowledge as a primary barrier, it may not be the most significant one.

While less commonly cited than student lack of knowledge and student ideology, intrapersonal factors, fear of conflict and fear of rocking the boat, were also identified as barriers by a number of institutions. However, the numbers are surprisingly low. This may be because professors and ministers underestimate the level of conflict students face regarding evolution (Barnes and Brownell 2016), fail to acknowledge their own fears about teaching evolution, or legitimately have no concerns about potential conflict within themselves or their students. Faculty falling into the last category may be overrepresented in our sample, since they signed up to come to the workshop and may therefore feel more comfortable with the topic of evolution, i.e., have less intrapersonal conflict. Previous research has suggested that fear of conflict and fear of rocking the boat are indeed significant barriers within the general population of teachers. Hawley and Sinatra (2019) found in an open discussion with teachers about their anxieties regarding teaching evolution that many teachers expressed fear of backlash from the community, administrative consequences, ostracism, and more.

Fortunately, there is an ample amount of literature demonstrating that faculty can address student fear surrounding evolution (Manwaring et al. 2015; Barnes and Brownell 2016; Lindsay et al. 2019; Tolman et al. 2020; Barnes and Brownell 2017; Truong et al. 2018; Bertka et al. 2019), and can increase student knowledge of evolutionary theory (Sinatra et al. 2003; Mead et al. 2017; Walter et al. 2013; Rios et al. 2015; Dunk et al. 2019), which account for many of the reported internal factors.

### External factors

Not surprisingly, external factors fit into the outer levels of the ecological model including institutional factors, community factors, and even public policy. The results show that, of the external factors, the institutional factor of a lack of discourse around science and faith was most commonly mentioned. According to previous research, many instructors do not have adequate training or knowledge to discuss religion when teaching evolution, and thus spend less time addressing it (Barnes and Brownell 2016). This leads to a lack of conversation around evolution within the classroom if students are highly resistant due to religious reasons. Nehm and Schonfeld (2007) found that even with adequate knowledge about evolution, many instructors still prefer not to teach it. In their study, instructors attended a workshop to address teachers’ misconceptions with evolution. After the workshop, instructors had a significant increase in their knowledge of evolution, but not an increase in their desire to teach evolution. When instructors consciously make a decision to limit the teaching of evolution, an environment is created that leads to less discourse around science and faith in the classroom.

Additionally, many institutions listed politics as a concern, another institutional factor. Faculty and students may fear that the teaching of evolution may be incompatible with governmental, institutional, or religious views. Many students at religious institutions may believe that their church’s stance is incompatible with evolution, a community factor. In some religions, religious leaders have issued statements against evolution, causing students to reject the teachings of evolution further (Coyne 2012). Being a part of a religious affiliation that does not openly reject evolution may help students and faculty be more open to discussing evolution in the classroom. Furthermore, departmental culture and politics can obstruct professors from improving their teaching methods in general, including evolution units specifically. Many professors report a lack of administrative support as a barrier to improving their teaching; they may fear decreased ratings and lost opportunities for tenure if they explore different ways to teach evolution that end poorly (Sunal et al. 2001; Brownell and Tanner 2012). Brownell and Tanner (2012) have proposed that this may be compounded by the prevailing culture within academia that emphasizes research over pedagogy. Professors may feel pressure to publish at the expense of the quality of their teaching or may even feel that teaching is the less significant aspect of their occupation (Brownell and Tanner 2012). Developing faculty learning communities to facilitate student-centered learning at the departmental level could be an important step in improving biology education generally, and the teaching of evolution specifically (Elliott et al. 2016). Many professors, however, also fear political backlash at the community level (Hawley and Sinatra 2019). While improving departmental culture may not directly be able to offset all aspects of political backlash, it may be a vital step in arming professors with the knowledge and confidence they need to develop effective evolution units within their courses.

Another institutional barrier when teaching evolution is the mixed messages to students. This could stem from issues within the curriculum, or even with intrapersonal conflict within faculty. According to previous research, instructors at more religious institutions may not personally believe their religious beliefs are compatible with evolution, thus leading to a lack of teaching (Barnes and Brownell 2016). Additionally, many of these instructors believe that the religious beliefs of the students may not be compatible with evolution, and thus they shy away from teaching evolution altogether (Barnes and Brownell 2016). Another study examined undergraduate biology instructors and found that in order to maintain their high level of professional identity, many instructors preferred to focus on research rather than teaching (Brownell and Tanner 2012). Instructors were less likely to change the way they taught, even in light of new teaching methods.

Students may also receive mixed messages during instruction from professors who differ in opinion. We have found anecdotally that many students have religion instructors who are anti-evolution, but biology instructors who are pro-evolution—students may then be left with mixed messages from professors regarding evolutionary theory. Lack of faculty involvement and collaboration could lead to many inconsistencies within teaching evolution and mixed messages to students.

Lastly, a fair number of institutions cited demographics as a concern, a factor exacerbated by the different community factors that would accompany such demographics. The diversity of religious beliefs was the main demographic concern when it comes to evolution discourse in the classroom. There is great diversity among Christian denominations and compatibility with evolution and their faith. The theology of denominations such as Roman Catholic, United Methodist, Evangelical Lutheran Church of America, and the Presbyterian Church is generally seen as compatible with evolutionary theory, whereas the Southern Baptist, International Circle of Faith, and Seventh Day Adventists theologies are not. Many other denominations’ positions on compatibility of evolution is unclear (Martin and Compatibility of Major U.S. 2010). But differences in beliefs were stated clearly by many leaders of each church specifically listed above. For instance, the Archbishop Gianfranco Ravasi, from the Roman Catholic church, stated, “What we mean by evolution is the world as created by God” (Strickland 2009). This statement is radically different from what the president of the Southern Baptist Theological Seminary stated. He said, “Evangelical Christianity and evolution are incompatible beliefs that cannot be held together logically within a distinctly Christian worldview” (Elliott et al. 2016). These extreme differences can raise a challenge as instructors are developing a curriculum for students of various beliefs, even if they are all Christian.

Barnes and Brownell (2018) examined the difference between the religiosity of instructors and their students, and how this played a role in teaching evolution A significantly higher percent of students claimed to have religious beliefs compared to the instructors (Barnes and Brownell 2018). Many religious students assumed that their instructor was not accepting of their religious beliefs. Winslow et al. (2011) found that students at various Christian universities were in the process of reconciling their religious beliefs with evolution. Instructors who were more open to discuss religion in the context of evolution were able to help students better reconcile their beliefs. It is important for instructors to recognize the religious diversity within the classroom and create an environment where students with both secular and religious views can feel comfortable learning evolution.

While educators have little sway over institutional, governmental, and theological politics, and cannot change the student demographics, they can influence discourse (Sunal et al. 2001; Kreber 2004,2005), which could logically reduce mixed messaging to students.

### Resources

Resources more commonly suggested by the professors and ministers included both interpersonal and institutional factors including curriculum, faculty as a force for change, and resources to negate conflict. These resources were followed by assets and collaboration with other institutions (Fig. 4). As noted in “Results” section, curriculum, an institutional factor, was the most commonly suggested resource. Currently, there are many different studies that explore unique ways to develop an evolution curriculum in higher education. Educating students about the nature of science has been shown to increase the acceptance of evolution (Cofré et al. 2018). Professors can enhance their evolution instruction by teaching students the nature of science before evolution units are taught (Nelson et al. 2019). They can also use a reconciliation model when teaching evolution to their students—this would not only change their curriculum, but will also help negate conflict (Barnes and Brownell 2016, 2017). In one study, four religious institutions presented potential compatibility between religion and evolution. The information presented on compatibility was affiliated with the university. Students showed significant gains in acceptance of evolution and did not dismiss their faith (Lindsay et al. 2019). This intentional instruction can help students reconcile their beliefs with the theory of evolution. In 2017, Barnes et al. (2017b) created a different reconciliation model at a public university that included readings on the compatibility of religion and evolution, timeline activities, evaluation of sources, and role models. This evolution module reduced the number of students who perceived conflict between evolution and religion by half (from 50 to 26%). With evidence from these studies, and many others, curriculum is one of the most important resources that schools need to nurture healthy discourse around difficult issues. Many professors indicated this on their posters, demonstrating their belief that curriculum can have a positive impact.

Another resource that was deemed as helpful was the interpersonal factor, faculty as a force for change. A network of faculty (within or outside of an institution) has been shown to be helpful (Sunal et al. 2001). Professors want to feel like they are making a difference and are able to overcome barriers as they work with colleagues. One way the professors can collaborate in regard to evolution is through faculty learning communities (FLC). FLCs have been shown to maintain the individual autonomy of professors, while also fostering collaboration and leading to an increase in student learning (Elliott et al. 2016). These communities could be helpful for professors to not only collaborate within their own department, but across disciplines (i.e., biology and religion departments). FLCs can be utilized with multiple institutions, but further research is required to understand how this would work in the context of evolution education. Curricula could be developed among multiple institutions in a similar format as FLCs.

While there is not much research on collaboration with other institutions, there is research that shows how beneficial it is to have a positive role model for students. Role models can help with negating conflict but could also be used in collaboration with other institutions to enhance learning. In one study, two guest speakers spoke: a devout Catholic male, who is a public defender of evolution, shared his own journey of reconciling science with evolution; the other speaker was a female ecologist and evolutionary biologist. She presented her work with microbial communities to showcase current research with evolution. These role models helped to provide possible positive role models for students to connect with and see that they can hold religious beliefs and defend evolution (Truong et al. 2018). Role models could also be faculty who help create a force for change. Holt et al. (2018) found that a role model had the biggest impact with helping students reconcile evolution and religion. Another study surveyed students through essays and found that they liked the authenticity and transparency of their professors who were straightforward in communicating their views on evolution and their religious beliefs, which created a positive relationship between science and religion (Winslow et al. 2011). Faculty can be a force for change by encouraging critical thinking, that will lead to transformative learning. This will help students challenge and grapple with the ideas that have been presented to them in their homes, school, and social life. Once students are able to engage with their thoughts, they are able to see other perspectives and reconstruct their own sense of self (Quinlan 2016). This idea of nurturing students with developing selves was found to be crucial for instructional success in a secondary science class, in regard to evolution (Scharmann and Grauer 2020). Other ways professors can have a positive effect on students while teaching evolution includes not forcing students to accept evolution and respecting students’ multiple viewpoints (Truong et al. 2018). Professors can be a force for change; students look up to their professors.

Curriculum and assets, which are both institutional factors and were commonly listed resources, are externally providable through grants and curriculum made publicly available. These resources can be procured much more rapidly than the cultural change necessary to help faculty be a force for change (Sunal et al. 2001) and the development of resources for negating conflict such as visiting authorities, or change in theology to a stance that is more accepting of science in general, and evolution in particular.

### Limitations

It is important to note that this study is qualitative in nature. While we include quantification, these are meant solely to help readers visualize what our respondents were saying. These numbers are not meant to imply that the responses of workshop participants are representative of science educators nationwide. Further research with a larger sample size, and more random sampling is needed to make these claims. Indeed, we call for further work to be explored in this area.

## Conclusions

We believe that public acceptance of evolution is of utmost importance. To quote Dobzhansky (1973), “Nothing in Biology makes sense except in the light of evolution” (p. 125). Because evolution is the unifying theory of biology it is reasonable to conclude that decisions and beliefs surrounding topics such as vaccines, antibiotic resistant bacteria, and the current biodiversity crisis cannot be fully formed without an acceptance of evolutionary theory. Indeed, research suggests that students who understand, but do not accept, evolution do not apply evolutionary thinking when making public decisions related to conservation (Sinatra et al. 2003; Rosengren et al. 2012). Recently, experts have argued that, amidst the recent outbreak of the virus SARS-CoV-19, “decisions about surveillance, monitoring, containment, and vaccine and drug development will be immeasurably more efficient and effective if basic principles of evolution are taken into account” (Reid 2020).

While public schools could potentially be a means for increasing acceptance of evolution, they are, as a whole, not adequately addressing this subject. After nearly a century, the fight over teaching creationism in public schools is not going away (Hall and Woika 2018). Many science teachers are unsure about teaching evolution in their classrooms, and even believe creationism should be taught as an alternative to evolutionary theory (Nehm and Schonfeld 2007). In 15% of the most conservative school districts in America, 40% of Biology teachers do not accept evolution, and do not devote time to the subject; many other teachers do not teach evolutionary theory because they fear controversy (Berkman and Plutzer 2011). A number of teachers who do teach evolution downplay controversy, which limits student comprehension of the evidence and scientific consensus behind the theory of evolution (Berkman and Plutzer 2012). Data from pre-service teachers is also discouraging. Factors that influence acceptance of evolution amongst the general public (e.g. religiosity, and an understanding of the nature of science and the facts of evolution) also influence pre-service teachers (Glaze et al. 2015). As with many practicing teachers, a number of pre-service teachers do not accept evolution as scientific fact, and are not planning on teaching it (Balgopal 2014). Hesitancy of teachers to teach evolution is not a phenomenon contained within the United States. To our knowledge, it has also been documented in Egypt (Mansour 2008), South Korea (Kim and Nehm 2011), Brazil, Argentina and Uruguay (Silva et al. 2021), as well.

Unlike public school teachers, university faculty are highly accepting of evolutionary theory (Rice et al. 2015), and thus offer a more immediate solution to low public levels of evolution acceptance. This places professors in an advantageous position to employ the tools known to increase undergraduate acceptance of evolution: namely teaching students to reconcile evolution with their religious faith, teaching students the nature of science and teaching about the mechanics of evolution (Lindsay et al. 2019). Those faculty that are religious, or are teaching at a religious institution, also have the potential to be role models that provide a pathway for religious students to accept evolution, a factor known to lead to greater acceptance of evolutionary theory (Holt et al. 2018). Additionally, instructors at religiously affiliated institutions have the added benefit of being in the position to employ religious cultural competence in evolution education (ReCCEE), an important part of the reconciliatory approach known to lead to gains in undergraduate acceptance of evolution (Barnes and Brownell 2016; Lindsay et al. 2019; Tolman et al. 2020). Despite their advantageous position, many professors are not comfortable employing strategies such as a reconciliatory approach, or do not feel that it is their purpose to help their students accept evolution (Barnes and Brownell 2016). It is critical to understand what factors influence the discourse around faith and science, the barriers professors face when teaching evolution, and the resources they feel would be helpful to ensure that university faculty are using their position to increase evolution acceptance. Using an ecological model as a framework (Sallis et al. 2008) can help us understand the complex environment in which faculty find themselves and consequently the factors that are exerting influence over their decisions regarding the teaching of evolution.

This study is a great starting point in identifying barriers at each level of the ecological framework, but we acknowledge that all of our participants elected to come to a workshop centered around helping faculty develop a reconciliatory model to increase undergraduate acceptance of evolution in their classrooms. Participation in this workshop could indicate that our sample was more enthusiastic about the idea of increasing evolution acceptance amongst their students (i.e., they lacked certain intrapersonal factors that others might face), and backed by more supportive administrations (i.e., they faced less institutional-level barriers), than university faculty as a whole. We call for broader research into this issue to gain a greater understanding of barriers to teaching evolution in higher education. Doing so could be an important means of unlocking the potential of higher education to influence public acceptance of evolution and address the problem of scientific illiteracy as a whole.

## Supplementary Information

**Additional file 1. **Photographs of Posters.

## Data Availability

Reconciliatory curricular materials created by teams who participated in the workshop can be found at https://biology.byu.edu/reconciling-evolution. Data was harvested from posters. Photographs of the posters are available in Additional file 1.

## Abbreviations

ReCCEE: Religious cultural competence in evolution education
FLC: Faculty learning communities

## References

[CR1] Alters BJ, Alters S (2001). Defending evolution in the classroom: a guide to the creation/evolution controversy.

[CR2] Augustine N (1998). What we don’t know does hurt us. How scientific illiteracy hobbles society. Science.

[CR3] Baker JO (2013). Acceptance of evolution and support for teaching creationism in public schools: the conditional impact of educational attainment. J Sci Study Relig.

[CR4] Balgopal MM (2014). Learning and intending to teach evolution: concerns of pre-service biology teachers. Res Sci Educ.

[CR5] Barnes ME, Brownell SE (2016). Practices and perspectives of college instructors on addressing religious beliefs when teaching evolution. CBE Life Sci Educ.

[CR6] Barnes ME, Brownell SE (2017). A call to use cultural competence when teaching evolution to religious college students: introducing religious cultural competence in evolution education (ReCCEE). CBE Life Sci Educ.

[CR7] Barnes ME, Brownell SE (2018). Experiences and practices of evolution instructors at Christian universities that can inform culturally competent evolution education. Sci Educ.

[CR8] Barnes ME, Truong JM, Brownell SE (2017). Experiences of Judeo-Christian students in undergraduate biology. LSE.

[CR9] Barnes ME, Elser J, Brownell S (2017). Impact of a short evolution module on students’ perceived conflict between religion and evolution. Am Biol Teach.

[CR10] Berkman MB, Plutzer E (2011). Defeating creationism in the courtroom, but not in the classroom. Science.

[CR11] Berkman M, Plutzer E (2012). An evolving controversy: the struggle to teach science in science classes. Am Educ.

[CR12] Bertka CM, Pobiner B, Beardsley P, Watson WA (2019). Acknowledging students’ concerns about evolution: a proactive teaching strategy. Evol Educ Outreach.

[CR13] Brownell SE, Tanner KD (2012). Barriers to faculty pedagogical change: lack of training, time, incentives, and…tensions with professional identity?. CBE Life Sci Educ.

[CR14] Charmaz K (2014). Constructing grounded theory.

[CR15] Cofré HL, Santibáñez DP, Jiménez JP, Spotorno A, Carmona F, Navarrete K (2018). The effect of teaching the nature of science on students’ acceptance and understanding of evolution: myth or reality?. J Biol Educ.

[CR16] Coyne JA (2012). Science, religion, and society: the problem of evolution in America. Evolution.

[CR17] Dobzhansky T (1973). Nothing in biology makes sense except in the light of evolution. Am Biol Teach.

[CR18] Dunk RDP, Barnes ME, Reiss MJ, Alters B, Asghar A, Carter BE (2019). Evolution education is a complex landscape. Nat Ecol Evol.

[CR19] Elliott ER, Reason RD, Coffman CR, Gangloff EJ, Raker JR, Powell-Coffman JA (2016). Improved student learning through a faculty learning community: how faculty collaboration transformed a large-enrollment course from lecture to student centered. LSE.

[CR20] Evans JH (2011). Epistemological and moral conflict between religion and science. J Sci Study Relig.

[CR21] Glaze AL, Goldston MJ, Dantzler J (2015). Evolution in the southeastern usa: factors influencing acceptance and rejection in pre-service science teachers. Int J of Sci and Math Educ.

[CR22] Hall GE, Woika SA (2018). The fight to keep evolution out of schools: the law and classroom instruction. Am Biol Teach.

[CR23] Hawley PH, Sinatra GM (2019). Declawing the dinosaurs in the science classroom: reducing Christian teachers’ anxiety and increasing their efficacy for teaching evolution. J Res Sci Teach.

[CR24] Heddy BC, Nadelson LS (2012). A global perspective of the variables associated with acceptance of evolution. Evol Educ Outreach.

[CR25] Holt EA, Ogden TH, Durham SL (2018). The positive effect of role models in evolution instruction. Evol Educ Outreach.

[CR26] Inc G. Evolution, creationism, intelligent design. Gallup.com; 2007. https://news.gallup.com/poll/21814/Evolution-Creationism-Intelligent-Design.aspx. Accessed 18 Mar 2021.

[CR27] Inc G. In U.S., belief in creationist view of humans at new low. Gallup.com; 2017. https://news.gallup.com/poll/210956/belief-creationist-view-humans-new-low.aspx. Accessed 18 Mar 2021.

[CR28] Keeter S, Smith G, Masci D. The culture of science: how the public relates to science across the globe. Bauer MW, Shukla R, Allum N, editors. New York: Routledge; 2012. p. 336–52.

[CR29] Kim SY, Nehm RH (2011). A cross-cultural comparison of Korean and American science teachers’ views of evolution and the nature of science. Int J Sci Educ.

[CR30] Kreber C (2004). An analysis of two models of reflection and their implications for educational development. Int J Acad Dev.

[CR31] Kreber C (2005). Reflection on teaching and the scholarship of teaching: focus on science instructors. High Educ.

[CR32] Lindsay J, Arok A, Bybee SM, Cho W, Cordero AM, Ferguson DG (2019). Using a reconciliation module leads to large gains in evolution acceptance. LSE.

[CR33] Mansour N (2008). Religious beliefs: a hidden variable in the performance of science teachers in the classroom. Eur Educ Res J.

[CR34] Manwaring KF, Jensen JL, Gill RA, Bybee SM (2015). Influencing highly religious undergraduate perceptions of evolution: Mormons as a case study. Evol Educ Outreach.

[CR35] Martin JW (2010). Compatibility of major U.S. Christian denominations with evolution. Evol Educ Outreach.

[CR36] Mazur A (2004). Believers and disbelievers in evolution. Politics Life Sci.

[CR37] Mead R, Hejmadi M, Hurst LD (2017). Teaching genetics prior to teaching evolution improves evolution understanding but not acceptance. PLoS Biol.

[CR38] Miller KR (2008). Only a theory: evolution and the battle for America’s soul.

[CR39] Moore R, Cotner S (2009). The creationist down the hall: does it matter when teachers teach creationism?. Bioscience.

[CR40] Nadelson LS, Southerland S (2012). A more fine-grained measure of students’ acceptance of evolution: development of the inventory of student evolution acceptance—I-SEA. Int J Sci Educ.

[CR41] National Science Teaching Association. NSTA position statement: evolution. nsta.org. https://www.nsta.org/about/positions/evolution.aspx. Accessed 13 Apr 2020.

[CR42] Nehm RH, Schonfeld IS (2007). Does increasing biology teacher knowledge of evolution and the nature of science lead to greater preference for the teaching of evolution in schools?. J Sci Teach Educ.

[CR43] Nelson CE, Scharmann LC, Beard J, Flammer LI (2019). The nature of science as a foundation for fostering a better understanding of evolution. Evol Educ Outreach.

[CR44] Pew Research Center. Evolution and perceptions of scientific consensus. Pew Research Center Science & Society; 2015. https://www.pewresearch.org/science/2015/07/01/chapter-4-evolution-and-perceptions-of-scientific-consensus/. Accessed 21 Feb 2020.

[CR45] Pew Research Center. Exploring different ways of asking about evolution|Pew Research Center; 2019. https://www.pewforum.org/2019/02/06/the-evolution-of-pew-research-centers-survey-questions-about-the-origins-and-development-of-life-on-earth/. Accessed 9 July 2020.

[CR46] Quinlan KM (2016). How emotion matters in four key relationships in teaching and learning in higher education. Coll Teach.

[CR47] Reece JB, Urry LA, Cain ML, Wasserman SA, Minorsky PV, Jackson RB (2011). Campbell biology.

[CR48] Reid A. Evolution and epidemics. National Center for Science Education; 2020. https://ncse.ngo/evolution-and-epidemics. Accessed 13 Apr 2020.

[CR49] Rice JW, Clough MP, Olson JK, Adams DC, Colbert JT (2015). University faculty and their knowledge & acceptance of biological evolution. Evol Educ Outreach.

[CR50] Rios K, Cheng ZH, Totton RR, Shariff AF (2015). Negative stereotypes cause Christians to underperform in and disidentify with science. Soc Psychol Pers Sci.

[CR51] Rosengren KS, Brem SK, Evans EM, Sinatra GM (2012). Evolution challenges: integrating research and practice in teaching and learning about evolution p. 348.

[CR52] Sallis JF, Owen N, Fisher EB (2008). Ecological models of health behavior. Health behavior and health education: theory, research, and practice.

[CR53] Scharmann LC, Grauer BL (2020). Critical relationships in managing students’ emotional responses to science (and evolution) instruction. Evol Educ Outreach.

[CR54] Scott EC (2009). Evolution vs creationism: an introduction.

[CR55] Silva HM, Oliveira AW, Belloso GV, Díaz MA, Carvalho GS (2021). Biology teachers’ conceptions of Humankind Origin across secular and religious countries: an international comparison. Evol Educ Outreach.

[CR56] Sinatra GM, Southerland SA, McConaughy F, Demastes JW (2003). Intentions and beliefs in students’ understanding and acceptance of biological evolution. J Res Sci Teach.

[CR57] Strickland E. Vatican gives darwin a big birthday hug, leaving creationists on the fringes. Discover Magazine; 2009. https://www.discovermagazine.com/planet-earth/vatican-gives-darwin-a-big-birthday-hug-leaving-creationists-on-the-fringes. Accessed 19 Mar 2021.

[CR58] Sunal DW, Hodges J, Sunal CS, Whitaker KW, Freeman LM, Edwards L (2001). Teaching science in higher education: faculty professional development and barriers to change. Sch Sci Math.

[CR59] Tolman ER, Ferguson DG, Mann M, Cordero AM, Jensen JL (2020). Reconciling evolution: evidence from a biology and theology course. Evol Educ Outreach.

[CR60] Truong JM, Barnes ME, Brownell SE (2018). Can Six Minutes of culturally competent evolution education reduce students’ level of perceived conflict between evolution and religion?. Am Biol Teach.

[CR61] Walter EM, Halverson KM, Boyce CJ (2013). Investigating the relationship between college students’ acceptance of evolution and tree thinking understanding. Evol Educ Outreach.

[CR62] Winslow MW, Staver JR, Scharmann LC (2011). Evolution and personal religious belief: Christian university biology-related majors’ search for reconciliation. J Res Sci Teach.

