# Supplementary material for: Barriers to teaching evolution in higher education
Source: Evolution (N Y). 2021 Aug 13;14(1):12. doi: 10.1186/s12052-021-00151-1 (PMC8360818; doi:10.1186/s12052-021-00151-1)
Supplement: Supplementary file 1 — Additional file 1. Photographs of Posters. [file 12052_2021_151_MOESM1_ESM.pdf]

Open to Discourse
But not a Priority

Resources: a core group of Science and faith faculty that plan convocations, etc. Links on College of Sciences + Mathematics website

Curricular
Resources
General Ed opportunities to address - 1st Year "Ways of Knowing"
- 3rd Year "Faith + Science" Courses
- Understanding the Bible
- Creation + Evolution

[redacted]

V.P. for Spiritual Development has budget to bring Science + Faith Speakers to campus twice annually

Challenge: Most Students are taught Evolution by adjunct Instructors with no Standard evolution Curriculum

Challenge: Epistemological Relativism of "All" Students - Religious or Agnostic
"It's my opinion... You can't deny it."

Challenge: Dominant Hermeneutic Presumption - again across religious + non-religious students
Serious = Literal interpretation of Bible
Dismissive = Symbolic Interpretation of Bible

Diverse student body re: Spiritual Dispositions
- ~1/3 Faith is enriching
- ~1/3 Faith is stifling
- ~1/3 Tell me something interesting, and I'll listen

Forum for Dialogue

[redacted]

1. Discussed informally among students and faculty, some deliberate discussions in Biology + Religion courses. Some concerns over adjunct professors.

2. Cultural inertia and politics; Lay ministry; Political tendencies.

3. Cultural inertia, misinterpretation of the teachings about the Creation, human origins and relationship between evolution and "creation" of humans.

4. Faculty in religion and science; accepting/supportive admin.

5. Admin, Science and religion forums/courses, unified message ~~of~~ related to evolution/science and the Gospel. Currulular alighment.

[redacted]

State of Discourse - Emerging, more on the academic side, less ~~on~~ with denominational leadership

Internal + External Factors - High level of trust with the institution - External: Denomiation conflates "evolution" with Atheistic Evolution. Rules out theistic evolution.

Reconciliation Challenges

External stakeholders, denominational leadership + pastors. (Politics + Perceived Conflict), fear, Ingroup/Outgroup

Discourse Resources

Theology, Philosophy, Science coursework. Esp. Hermeneutics + nature of science.

Add'l Resources

Rau, Mapping the Origins Debate

[redacted]

1) high school juniors and senior students: Warfare model is in play
secular schools: fearful of religious encroachment
religious schools: fearful of undermining Scriptural authority.

2) There is historically a wider tension in Israeli society between ~~the~~ secular and religious Jews. The discourse in Israel is not only about students being able to keep their faith; it is about a social divide between the secular and religious communities. Even in the religious schools, the secular curriculum is totally distinct from the religious curriculum (stratified), so students will later go in one direction and secularize or

3) The main problem has to do with the <u>politics</u> of the Ministry of Education and in Israeli society generally.

4) Midrash – the recognition that any text can be interpreted in multiple ways – is inherent in the Jewish tradition. There are also strong and varied Jewish theological resources (~~or~~ rationalist and Kabbalist) that support reconciling religious texts with science. That said, there are some Orthodox rabbis and other Jews who see evolution as being in conflict with Judaism.

5) ~~It~~ Templeton just gave Haifa University money to help build a team of people to ~~help~~ ~~[illegible]~~ conduct research on the teaching of evolution in Israeli schools, to prepare curricular materials, and ~~to~~ also teacher education materials. Israel in ~~their~~ its new natural history museum in Tel Aviv should create a Broader Social Impacts Committee, similar to the one at the Smithsonian.

[redacted]

1) graduate professional programs: not covered
undergraduate: skirted – only what students need to know for exams (MCAT, etc.)

2) Graduate programs: faculty feels they have no time to teach evolution; they must cover ~~the~~ what the curriculum requires
~~Undergraduate programs~~ – graduate faculty is non-denominational
Undergraduate program: too much of a hot potato – the faculty in the religious undergraduate program is Orthodox; in the heterogenious undergraduate program, faculty is also heterogenious, and evolution may be taught (not clear)

3) Touro is 45 years old. As it matures, it will need to address this issue more thoroughly, given that it was founded on Jewish principles.

4) A good relationship and mutual respect between the experts in the religious disciplines and the scientific disciplines. Moreover, today Touro has faculty members who are both scientists and religious.

5) Internally, the creation of a formal infrastructure to address these issues. Externally, to build upon the efforts of this group of scholars ~~to~~ in Utah to network and work together.

[redacted]

Presbyterian, but with students from many faith traditions

CORE ISSUE: Dispelling false dichotomy of

SCIENCE VS. RELIGION

No!!!

PROBLEM: STUDENTS ARRIVE AT COLLEGE WITH GAPS & MISCONCEPTIONS DUE TO INADEQUATE EDUCATION IN BOTH SCIENCE & FAITH

SOLUTION: TEACH HOW TO THINK, NOT WHAT TO THINK

- College is where you learn, surprisingly, what you don't know.
- Show that we don't yet have all the answers (in both faith and science) but "we're working on it" by asking questions and discussing ideas in open, welcoming setting

OUR APPROACH: Since we can't deal with all student Qs in classes (and students see that as professors' turf), we ha open lunch table for BIG QUESTIONS, especially those dealing with intersection of faith & science

Students are free to ask any questions and present their views i a welcoming, non-threatening forum.

This is what we feel education is all about!

General State:
healthy but minimal. Faith discussion?

Influencing factors:
BIG ONE: what they brought in w/them.
faculty input also influences

Challenges:
- Conservative church input
- Myths about science and what ev actually ~~is~~ claims
- Ignorance of biblical genres

Resources:
- Professor's open doors
- Books, websites

Additional resources:
Visiting authorities, special events
student clubs/interest

[redacted]

[redacted]

- State of discourse (faculty, students, classroom)
  - => Unless issues are raised, no active discourse
    - ↳ "don't ask, don't tell"

- Internal + external factors
  - => Students + faculty operate out of perceived conflict between faith + science

- Greatest challenges to reconciliation
  - => FEAR OF COMPROMISE
    - i) Biblical authority
    - ii) Cultural authority
    - iii) Religious identity

- Resources @ institution to nurture discourse:
  - i) King Institute for Faith + Culture
  - ii) Fall Faculty Workshops
  - iii) RELG 1001 - Foundation of Christian Thought and practice course required of all King students (graduating requirement)

- Other internal/external resources:
  - i) Proximity to Dayton, TN (site of Scopes Trial)
  - ii) Workshops for area pastors
  - iii) Proximity to Creation Museum (fostering dialogue)

1. friendly - apathetic

[illegible]. Catholic - Jesuit.

3. wonder → both understanding and acceptance

4. Cross-disciplinary Conversations/Mission and Identity Office

5. JTF Grants - university - school - department resources

[redacted]

[redacted] Secular, Heterogeneous Student Body

1 - Evolution + Religion recurrent discourse within + among Faculty, students, departments

2 - Diverse beliefs in faculty, student body. Culture + faith identities are strong despite secular Institution

3 - No institutional statement on evol./religion or plans for one

4 - No institutional resources

5 - there is a need for new discourse including books + materials. Perhaps external partnership w/ more experienced institution.

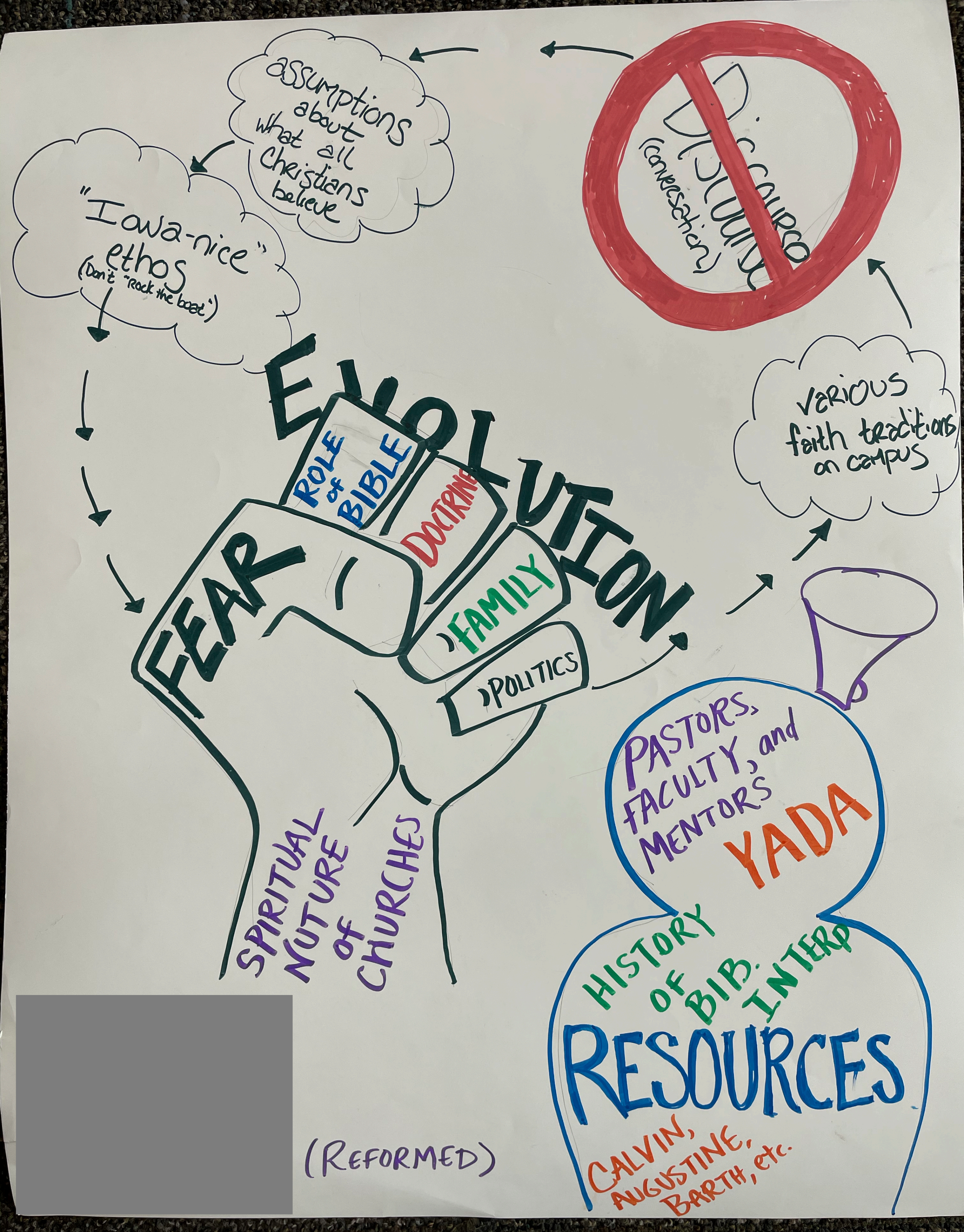
assumptions about what all Christians believe
"Iowa-nice" ethos
(Don't "rock the boat")
DISCOURSE
(conversation)
EVOLUTION
ROLE of BIBLE
DOCTRINE
FEAR
FAMILY
POLITICS
various faith traditions on campus
SPIRITUAL NUTURE of CHURCHES
PASTORS, FACULTY, and MENTORS
YADA
HISTORY of BIB. INTERP
RESOURCES
CALVIN, AUGUSTINE, BARTH, etc.
(REFORMED)

- LIMITED PUBLIC DISCOURSE
- CONSTRAINED BY A FUNDEMENTALIST READING OF SCRIPTURE
- FEAR IS A GREAT CHALLENGE TO ANY RECONCILIATION. FEAR OF LOSING FAITH OR ONE'S JOB.
- WE ARE A SMALL, RELATIONAL CAMPUS WHICH PROVIDES OPPORTUNITY FOR HEALTHY DIALOGUE.

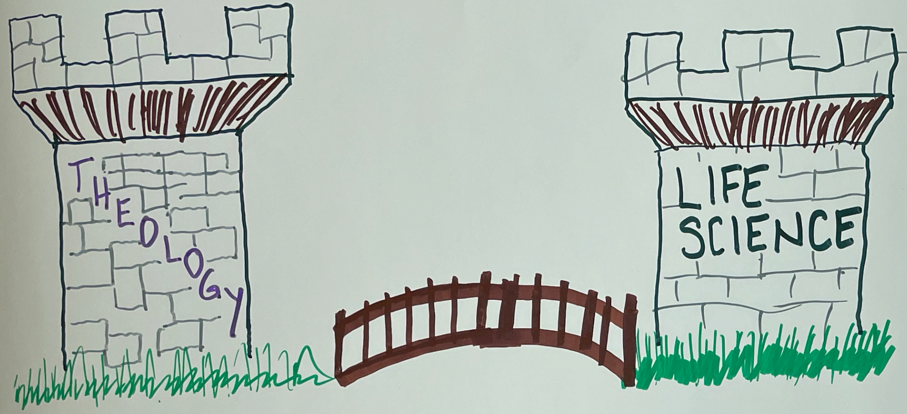

- Disciplines in Silos
- Theology Faculty lacks intellectual Curiosity
- No resistence to evolution on part of Admin.
- Bio faculty in agreement on including evolution in curriculum.

[redacted]

General State:

Faculty: open, accepting, no observed tension

Students: fear, curiosity, initial openness, vagueness

Influencing Factors

Internal: majority of faculty + students are members of the Church of Jesus Christ of Latter-day Saints; neither faculty nor admin are opposed to teaching evolution; cultural/religious homogeneity

External: Adoption of values of Church of Jesus Christ; integration of Institute instruction, cultural, regional, + economic diversity

Challenges:

A. Lack of diversity among students

B. Not enough dialogue among students + faculty

C. No data as to where students/faculty are on this discussion

Healthy Discussion Resources

A. Faculty

B. Students

C. Small classes, lots of connection among students/staff

Supporting Resources:

A. Money

B. Faculty buy-in on the importance of this reconciliation project

C. Faculty talks (formal/informal) on their personal faith + scholarly journey

Reality?

Misconceptions

- What science is → PROGRESS = Good → Perfectionism → Oh hey religion
- What religion is → REGRESS = BAD → Backwards & Stuck

- Evolution as progress

- Science as objective and pure knowledge and detached from social influence

- Most faculty membres accept theistic evolution
- A big portion of students were taught creationism at home / church

  → Evolution is taught in biology courses

- - This topic is sensitive to the relationships between students and parents
  - Compartmentalization among departments
  - Need deep theology in the topic of theistic evolution (for instance, eschatology)

- People

  Liberal arts mission statement → push us to have conversations

- Resources for parents and pastors

  A co-written book from a scientist and a theologian.
